# NMR-Based Metabolomics of Blood Serum in Predicting Response to Induction Chemotherapy in Head and Neck Cancer—A Preliminary Approach

**DOI:** 10.3390/ijms25147555

**Published:** 2024-07-10

**Authors:** Łukasz Boguszewicz, Agata Bieleń, Jarosław Dawid Jarczewski, Mateusz Ciszek, Agnieszka Skorupa, Jolanta Mrochem-Kwarciak, Krzysztof Składowski, Maria Sokół

**Affiliations:** 1 Department of Medical Physics, Maria Sklodowska-Curie National Research Institute of Oncology, Gliwice Branch, 44-102 Gliwice, Poland; mateusz.ciszek@gliwice.nio.gov.pl (M.C.); agnieszka.skorupa@gliwice.nio.gov.pl (A.S.); maria.sokol@gliwice.nio.gov.pl (M.S.); 2 1st Radiation and Clinical Oncology Department, Maria Sklodowska-Curie National Research Institute of Oncology, Gliwice Branch, 44-102 Gliwice, Poland; agata.bielen@gliwice.nio.gov.pl (A.B.); krzysztof.skladowski@gliwice.nio.gov.pl (K.S.); 3 Radiology and Diagnostic Imaging Department, Maria Sklodowska-Curie National Research Institute of Oncology, Gliwice Branch, 44-102 Gliwice, Poland; jaroslaw.dawid.jarczewski@gmail.com; 4 Analytics and Clinical Biochemistry Department, Maria Sklodowska-Curie National Research Institute of Oncology Gliwice Branch, 44-102 Gliwice, Poland; jolanta.mrochem-kwarciak@gliwice.nio.gov.pl

**Keywords:** head and neck cancer, induction chemotherapy, NMR, blood serum metabolomics, treatment prediction, personalized medicine

## Abstract

The role of induction chemotherapy (iCHT) in locally advanced head and neck squamous cell carcinoma (LA-HNSCC) is still to be established due to high toxicity and variable response rates. The aim of this retrospective study is to use NMR-based serum metabolomics to predict the response rates to iCHT from the pretreatment samples. The studied group consisted of 46 LA-HNSCC patients treated with iCHT. The response to the treatment was evaluated by the clinical, fiberoptic, and radiological examinations made before and after iCHT. The proton nuclear magnetic resonance (1H NMR) serum spectra of the samples collected before iCHT were acquired with a 400 MHz spectrometer and were analyzed using multivariate and univariate statistical methods. A significant multivariate model was obtained only for the male patients. The treatment-responsive men with >75% primary tumor regression after iCHT showed pretreatment elevated levels of isoleucine, alanine, glycine, tyrosine, N-acetylcysteine, and the lipid compounds, as well as decreased levels of acetate, glutamate, formate, and ketone bodies compared to those who did not respond (regression of the primary tumor <75%). The results indicate that the nutritional status, capacity of the immune system, and the efficiency of metabolism related to protein synthesis may be prognostic factors for the response to induction chemotherapy in male HNSCC patients. However, larger studies are required that would validate the findings and could contribute to the development of more personalized treatment protocols for HNSCC patients.

## 1. Introduction

Organ preservation is the crucial goal of head and neck squamous cell carcinoma (HNSCC) treatment, with radio- (RT) or concurrent chemoradiotherapy (cCHRT) being the gold standard treatment modalities. However, in the advanced stages of the disease (locally advanced HNSCC, LA-HNSCC), RT and cCHRT may be inappropriate due to the large tumor volume and expected toxicity of high-volume radiotherapy. In such cases, a debulking or downstaging strategy is proposed before RT/cCHRT and is most often achieved by induction chemotherapy (iCHT) [1,2]. iCHT is beneficial for LA-HNSCC patients with multiple involved nodes or large-volume nodal disease [3]; however, it is not only burdened with relatively high toxicity [4,5,6], but also its effectiveness still needs improvement [7,8,9]. The complete clinical response (CCR, disappearance of target lesions) and partial response (PR, a reduction of at least 30% in the sum of the longest diameter of target lesions) to iCHT are observed in around 30 and 60% of LA-HNSCC patients, respectively, while in the remaining 30% of patients, the disease is stable or progressive. A partial response (PR) usually reaches up to or is slightly more than 60%, while the rest of the patients treated with iCHT have a progressive (PD, an increase of at least 20% in the sum of the longest diameter of the target lesions) or stable disease (SD, not eligible for PR or PD) [7,8,9]. Despite extensive research, the source of this variable individual response to iCHT in terms of tumor control and treatment-induced toxicity remains unknown, highlighting the strong need for new strategies to personalize treatment. The search for predictive biomarkers correlated with treatment response and/or toxicity is ongoing, but so far, none of them have even been tested in a clinical setting [10]. Early prediction of the treatment outcome or its toxicity may be highly beneficial for those who are at risk of developing severe toxicities or treatment failure—a different treatment strategy may be applied to these patients, avoiding unnecessary pain and/or increasing the chance of a better outcome. Reduced responsiveness of squamous cancer cells can arise from genetic, epigenetic, and microenvironmental factors. The most well-known mechanisms are drug inactivation, reduced intracellular drug accumulation, drug target alteration, activation of compensatory pathways for cell survival, regulation of DNA repair and cell death, tumor plasticity, and regulation of tumor microenvironments. As revealed in the meta-analysis of several randomized trials that examined iCHT-specific regimens, namely, TPF (docetaxel, cisplatin, and fluorouracil) versus PF (cisplatin plus fluorouracil), the results are inconclusive. In the case of the TPF regimen, a significant reduction in progression, locoregional failure, and distant failure were observed as compared to PF, while direct comparisons of induction chemotherapy regimens followed by chemotherapy versus chemotherapy alone in unresectable disease have not shown a significant difference in overall survival [11].

This preliminary research is consistent with the concept of personalized medicine—using 1H NMR spectroscopy, we analyzed the pretreatment metabolic profile of blood serum as a potential predictive biomarker of iCHT response. According to a recent literature review, NMR-based metabolomics has not yet been used for this purpose [10]; however, the results we previously obtained in a similar group of patients indicate that the NMR-detectable blood serum metabolites may have a predictive value in assessing the response to iCHT. As revealed from our previous study [12], the iCHT-induced increase in the blood serum lipids correlates significantly with the clinically and radiologically measured primary tumor shrinkage due to iCHT. This observation was exclusive to the male patients. This is especially important because men are at twofold to fourfold higher risk than women for developing HNSCC [13].

The aim of this study is to check whether the degree of radiological and clinical regression of the primary tumor and the stage of lymph node advancement can be concluded based on the metabolic profile obtained from the blood serum samples taken before iCHT.

## 2. Results

The detailed baseline characteristics of the patients under investigation are presented in Table 1.

### 2.1. Primary Tumor Regression Based on the Volumetric Measurements

In the study group, the median of the percentage of the primary tumor regression assessed by radiological imaging was 82.6% (ranged between 0 and 100%) as well as 90.6% (ranged between 12.5 and 100%) and 71.8% (ranged between 0 and 100%) for men and women, respectively. The difference between men and women is statistically significant (*p* = 0.037, MWU test). The prefix modifiers “c” and “y” describe the pre- (“c”) and post-iCHT (“y”) stages.

**Figure 1:**
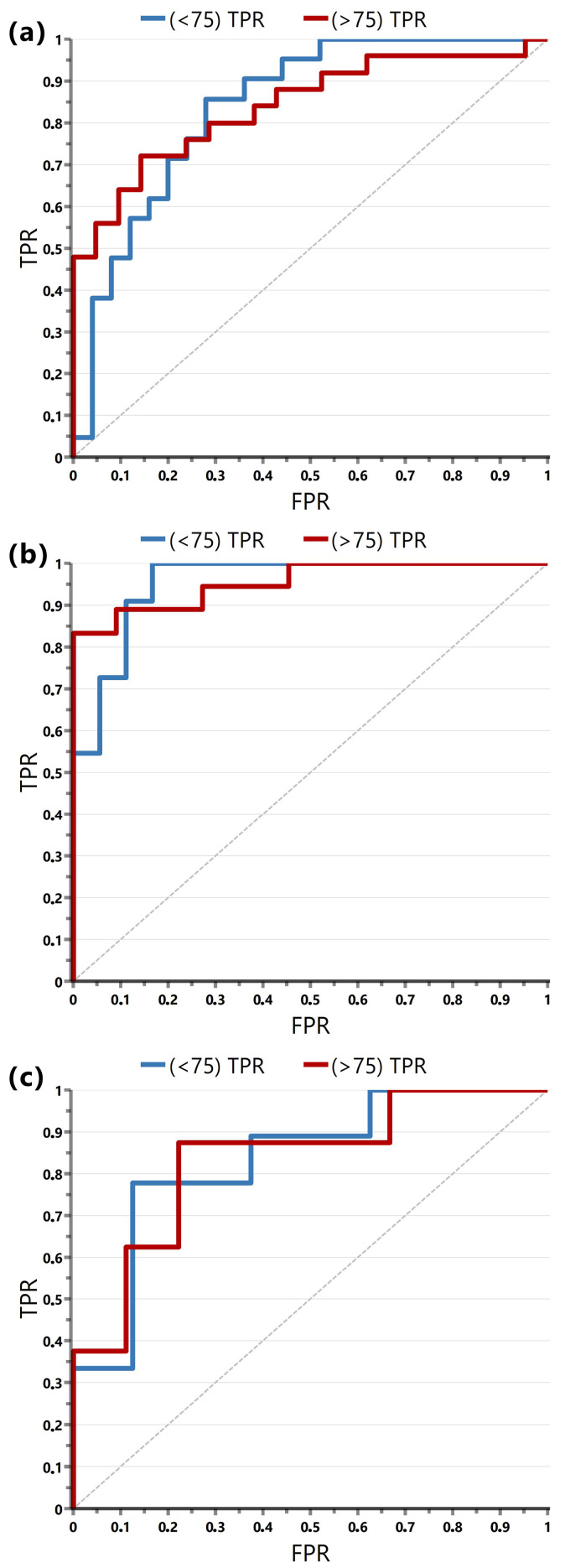
The ROC curves for the OPLS-DA models presented in Figure 2 and Table 2 show the true positive rate (TPR) against the false positive rate (FPR) for (**a**) the study group (the total area under the curve, AUC = 0.84), (**b**) the male group (AUC = 0.95), and (**c**) the female group (AUC = 0.83). The red and blue curves denote TPR for the responders (>75) and non-responders (<75), respectively. The TPR was calculated based on the OPLS-DA misclassification tables.

The patients were divided into two groups—the responders (the patients who benefit from iCHT) and the non-responders (those who do not benefit from iCHT)—based on the radiological regression of the primary tumor. Due to the small size of the study group, attempts to distinguish the responders from the non-responders based on the pretreatment metabolic profiles were performed for various primary tumor regression cut-off points, e.g., the median for each subgroup. A receiver operating characteristic (ROC) analysis was applied to select the optimal models possible and to discard the suboptimal ones. The best results (Figure 1) were obtained for a regression cut-off point of 75%, and this value was used in this work. Thus, the responders’ group involved patients who had at least a 75% radiological regression of the primary tumor after the iCHT, whereas in the non-responders, the radiological regression of the primary tumor was <75% after iCHT.

**Figure 2:**
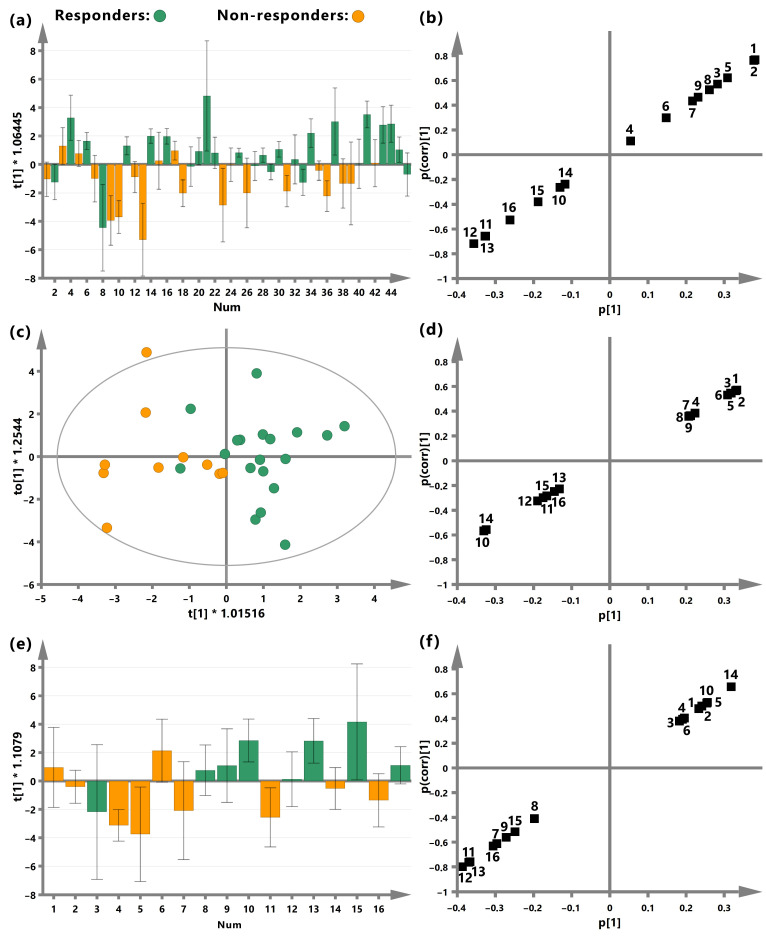
The results from the OPLS-DA analysis discriminating the iCHT responders from non-responders (the score plots: (**a**,**c**,**e**)) based on the pretreatment blood serum metabolites (the s-plots: (**b**,**d**,**f**)) for the study group (**a**,**b**), males (**c**,**d**), and females (**e**,**f**). The numbering of the metabolites on the s-plots corresponds with the numbering in Table 2: 1—isoleucine 0.95 ppm; 2—isoleucine 1.02 ppm; 3—alanine; 4—glycine; 5—tyrosine; 6—N-acetylcysteine; 7—lipids 0.9 ppm; 8—lipids 1.3 ppm; 9—lipids 5.3 ppm; 10—acetate; 11—glutamate; 12—3-hydroxybutyrate 4.21 ppm; 13—3-hydroxybutyrate 1.23 ppm; 14—formate; 15—acetone; 16—acetoacetate. The results take into account the resonance signals of the functional groups of a given metabolite at different chemical shifts, e.g., isoleucine at 0.95 and 1.02 ppm, lipids at 0.9, 1.3 and 5.3 ppm, as well as 3-hydroxyisobutyrate at 1.23 and 4.21 ppm.

**Figure 3:**
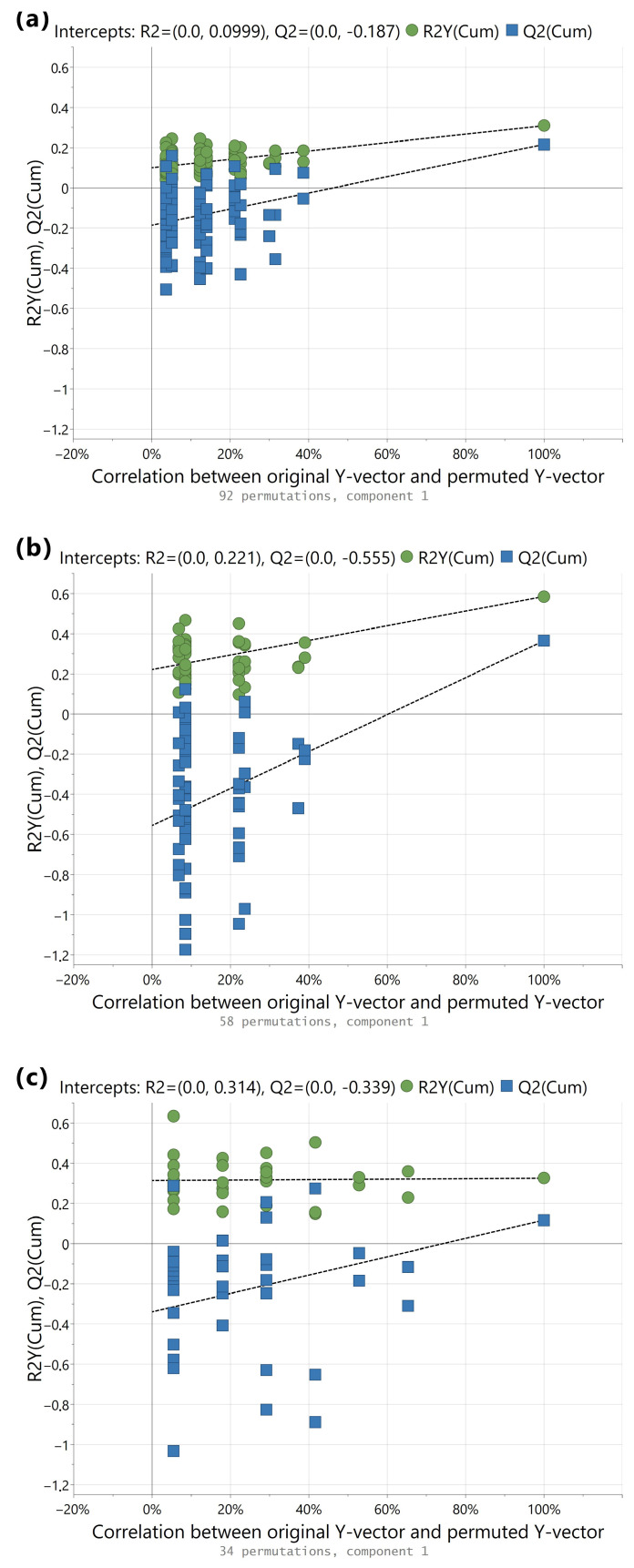
The results of permutation testing of the OPLS-DA models discriminating the responders from non-responders in the study group (**a**), males (**b**), and females (**c**). The number of permutations was set to twice the sample size.

Figure 2 shows the OPLS-DA results distinguishing the responders and the non-responders based on the pretreatment NMR blood serum metabolites. The summary of the OPLS-DA analysis is presented in Table 2. The results are presented for the study group (Figure 2a,b) as well as for the male (Figure 2c,d) and female (Figure 2e,f) groups separately. A decent separation with very few mixing samples is observed in the OPLS-DA score plots (Figure 2a,c,e); however, only the model built on the male group achieved a minimal statistical strength according to the OPLS-DA rules, i.e., Q2 > 0.3 and cv-ANOVA *p*-value < 0.05 (Table 2). Figure 2b,d,f shows the corresponding s-plots. The s-plots are used to visualize the intensity (horizontal direction) and reliability (vertical direction) of a biomarker. However, because the variables are UV-scaled, the information about intensity is lost, and only the reliability can be assessed from the s-plots. The metabolites with p(corr) > 0.3 (vertical direction) were selected as important for classification and are bolded in Table 2. The results of permutation testing of the OPLS-DA models is presented in Figure 3.

### 2.2. Complete Clinical Response (CCR) of the Primary Tumor and Complete Nodal Response (CNR)

In the study group, only three male patients achieved both CCR (yT = 0) and CNR (yN = 0) after iCHT. A total of 11 patients (10 males and 1 woman) achieved CCR only, and 17 patients (11 men and 6 women) achieved only CNR.

Two types of multivariate analyses were used in the CCR and CNR prediction trial: two-class OPLS-DA models distinguishing the patients with CCR (yT = 0) and CNR (yN = 0) from those without a complete regression (yT and yN > 0), and the regression PLS models correlating the yT and yN values with the individual metabolic levels. The analyses were performed for the entire group and by gender. Again, the best results were obtained for the male group, but this time, none of the multivariate models reached the significance threshold, and therefore, the summary of these results was omitted.

### 2.3. Metabolic Pathway Analysis

The low-molecular-weight metabolites listed in Table 2 were subjected to pathway analysis. The analysis revealed only one significantly enriched metabolic pathway (Holm-adjusted *p*-value < 0.05), the glyoxylate and dicarboxylate metabolism. There are 32 metabolites in total involved in this pathway; four of them were identified as important for discriminating the responders and non-responders: glycine, formate, acetate, and glutamate.

## 3. Discussion

In the present study, the pretreatment blood serum samples were used to distinguish the iCHT responders vs. non-responders. Two approaches were tested based on a percentage of radiological regression of the primary tumor and complete clinical regression (yT = 0 and/or yN = 0), respectively. The second approach yielded no statistically significant results. The division into the responders and non-responders (based on the radiological regression) was conducted by optimizing the ROC curve; finally, the cut-off point of 75% was chosen. The gold standard for assessing the treatment response in solid tumors is the so-called RECIST (response evaluation criteria in solid tumors) scale [14]. According to RECIST, a partial response is defined as at least a 30% decrease in the sum of the diameters of the target lesions, taking as reference the baseline sum diameters, while a complete response means a disappearance of all target lesions [14]; there is nothing in between in RECIST. A recent literature review on predictive biomarkers for the response of iCHT in HNSCC reveals that the RECIST guidelines are not strictly followed, and different cut-off points for the response are often chosen [10]. In general, patients with a tumor volume decrease of around 70% are the responders [10]; thus, the cut-off point chosen in this study seems justified.

As revealed from our previous studies, a molecular response to iCHT is gender-dependent. A common feature is an increase in the lipid signals after iCHT, but in the male group, an additional decrease in the glucose, alanine, and N-acetylglycoprotein (NAG, the NMR-detectable inflammatory marker) signals is observed [12]. However, most importantly, we have shown that, in men, the increase in the lipid signals and decrease in NAG after iCHT correlates with a clinical and radiological regression of the primary tumor [12], whereas no such correlation has been observed in women [12].

The present study yields similar results in terms of sex differences. This is visible in the parameters determining the quality of the OPLS-DA models, where the highest Q2 value = 0.36 (predictive ability of the model) and the cv-ANOVA *p*-value = 0.03 are observed for the male group. The OPLS-DA model involving the female data is not statistically significant (cv-ANOVA *p* = 0.42 and Q2 = 0.12), while that built on the entire group has a cv-ANOVA *p* < 0.05, but Q2 is below 0.3—the value taken as the lower threshold.

The succeeding discussion will, thus, focus on the metabolic profiles and their statistically significant features characterizing the response to iCHT. In the pretreatment samples from the male responders, the lipid signals from the methylene (-CH2-CH2-CH2-) and methyl (CH3-CH2-) groups at 1.3 and 0.9 ppm, respectively, and from the protons from the unsaturated fatty acid moieties (-CH=CH-) at 5.3 ppm, N-acetylcysteine, tyrosine, glycine, alanine, and isoleucine are increased, whereas those of acetate, glutamate, formate, and 3HB are lower than in the non-responders.

The above results indicate that the lipid profile of the blood serum is not only sensitive to chemotherapy, but it may also have a predictive value for the treatment’s effectiveness [15]. Although research on the impact of chemotherapy on the lipid profile has been conducted for many years [16,17], due to the complex feedback loops of the mechanisms regulating lipid homeostasis in cancer and during its treatment, the role of the lipid changes still remains virtually unknown [18]. Resolving these relationships may be crucial for the effective personalization of chemotherapy-based treatment, and this is also indicated by our research.

The metabolites that enable statistical identification of the male responders and non-responders are those involved in protein synthesis and degradation, i.e., alanine, glycine, glutamate, and isoleucine. Alanine is synthesized in the skeletal muscle from oxidation of the branched-chain amino acids (BCAAs: isoleucine, leucine, and valine) and transported to the liver, where it is catalyzed by alanine aminotransferase [19]. Alanine is also one of the major substrates for gluconeogenesis during glucose deficiency [19], while isoleucine stimulates glucose uptake in the skeletal muscle and depresses gluconeogenesis in the liver [20]. In patients with muscular dystrophies, the alanine levels were found to be reduced [21]. It was further noted that hepatic alanine catabolism, driven by chronic glucocorticoid and glucagon signaling, promotes hyperglycemia and skeletal muscle atrophy [22]. Glycine, an important inhibitory transmitter, among its other functions, is essential for protein synthesis and muscle growth [23,24].

We observed higher levels of glutamate—an intermediate of the TCA cycle—in the non-responders. Under normal conditions, the blood glutamate levels are maintained in a steady state, and a normal diet prevents significant fluctuations [25]. The literature data indicate that blood glutamate plays an important role in peripheral organs [26]. As revealed from the studies of the rate of glutamate uptake in various peripheral tissues and organs after intravascular injection of [14C]-Glu, the skeletal muscles contain the body’s largest storage pool of glutamate, accounting for approximately 59% of the total storage amount [27]. Glutamate is also found in the liver and kidneys, but when released by the liver, it is taken up by the muscles in the post-absorptive state and in a state of starvation in overweight people [28].

Based on the above, it can be assumed that the reduced levels of isoleucine, alanine, and glycine in the non-responders reflect their poorer muscular and/or nutritional (isoleucine is an essential amino acid, and glycine is not synthesized by humans to a sufficient extent) conditions compared to the responders. We tried to confirm this assumption by comparing the BMI (though this parameter does not reflect lean body mass well) as well as the albumin and prealbumin values in both groups: while the medians for all three parameters were higher in the responders, the difference, however, was not statistically significant.

The non-responders show lower levels of tyrosine as well as elevated acetate, 3HB, and, although with p(corr) < 0.3, acetone and AceAce. 3HB, AceAce, and acetone are the ketone bodies, usually elevated in blood in a carbohydrate-deficient state, and malnutrition [29]—the conditions that are prevalent in HNSCC patients [30]. Acetate is a fatty acid, structurally similar to the ketone bodies, and as the latter is the end product of fatty acid oxidation in the liver during fasting conditions, it is mainly utilized in the heart, skeletal muscle, and brown adipose tissue, acting as a nutrient deprivation and stress-indicator and as a substrate for protein and metabolite acetylation reactions [31]. Acetate is also a signaling and regulatory metabolite involved in inflammatory and stress responses and cancer metabolism [32]. The changes characteristic of malnutrition are not statistically significant, and the albumin values, although low, are within the reference range. However, considering that this is accompanied by disturbances in the amino acids related to the maintenance of the muscle tissue, the hypothesis of a worse nutritional status of the non-responders vs. responders should not be rejected.

Tyrosine is directly involved in protein phosphorylation and indirectly (through dopamine and catecholamines) in the regulation of immune response and neurotransmission [33,34]. Dopamine is required for numerous brain functions, whereas catecholamines are associated with the fight-or-flight response and prepare the body to deal with environmental stress [34]. Protein phosphorylation is important in cellular regulatory mechanisms such as protein synthesis, cell division and growth, and signal transduction [35]. Tyrosine phosphorylation activates, inter alia, p53 protein [35,36], which is presumably related to the response to chemotherapy, although it remains to be elucidated [10]. Tyrosine can be obtained from food or generated (in the liver) from phenylalanine through phenylalanine hydroxylase; its deficiency is strongly involved in the mechanisms of unipolar depression [37].

In our study, the non-responders showed elevated formate compared to the responders. Formate is an intermediate metabolite in one-carbon metabolism. It is produced from serine or glycine (this may be an additional factor contributing to the reduced glycine level in the non-responders) in the folate-dependent production process and folate-independent process from the oxidation of formaldehyde [38]. An increase in blood formate is observed in vitamin B12 deficiency, which, in turn, may be involved in head and neck carcinogenesis, as revealed by the study of Raval et al. [39]. Furthermore, an increased formate concentration within the tumor microenvironment can promote metastases [40], and such an increase was previously observed in rectal and esophageal cancer tissues [38].

Finally, the non-responders show lower NAC, N-acetyl derivative of the amino acid L-cysteine. NAC is a conditionally essential metabolite with relatively stable concentrations under healthy conditions [41]. It has been postulated that NAC promotes slow and sustained sulfane sulfur and hydrogen sulfide production, resulting in several cytoprotective effects, including stimulation of mitochondrial bioenergetics, modulation of protein function, as well as, which is especially important in HNSCC treatment, protection against irreversible oxidative damage and an increase in oxidant scavenging capacity [42]. The vast majority of available publications indicate the potentially protective effect of NAC in anticancer treatment and its ability to inhibit cancer cell proliferation, but there are also reports indicating no clinical translation of NAC [43,44]. Although NAC does not appear to affect overall survival in HNSCC [45], its higher concentrations may have a protective effect on the toxicity of chemo- and radiochemotherapy [46,47]. The assumption that the responders can better suppress oxidative stress is also supported by the increased glycine levels in this group. In addition to protein synthesis (discussed above), glycine, like NAC, has anti-oxidant properties [24,48,49].

The results of the metabolic pathway analysis revealed a significant enrichment of glyoxylate and dicarboxylate metabolism. Glyoxylate and dicarboxylate metabolism are associated with obesity, type 2 diabetes, and atherosclerosis cardiovascular disease [50]. Very recent findings identify it as the specific pathway and biomarker of oral squamous cell carcinoma cells in response to cisplatin [51] and the poor prognosis of HNSCC patients [52]. Although our research was conducted on a small group, the results obtained are supported by the available literature reports and may be an impulse to conduct further research using metabolomics based on NMR spectroscopy, which is a cheap, non-invasive, non-destructive, and highly repeatable research method.

### Limitations of the Study

This study was conducted on a quite small and gender-heterogeneous group, where women were underrepresented (29 men and 17 women). The difference in the statistical significance between males and females presumably results from the differences in the gender-specific sample sizes. On the other hand, the results obtained here are consistent with those obtained in a similar group of patients where we examined the molecular response to iCHT [12]. In our previous study, we identified a blood serum metabolic signature correlated with a radiological and clinical regression of the primary tumor, and this correlation was statistically significant only in male patients [12]. The biggest limitation related to the small group size is the lack of an external test set.

## 4. Materials and Methods

### 4.1. Characteristics of the Patient Group

The retrospective study was approved by the Ethics Committee and informed written consent was obtained from the participants. The studied group consisted of 46 LA-HNSCC patients, 29 men, and 17 women, all Caucasians, between 22 and 73 years (median 56 years). All patients were treated with radical intent in the 1st Radiation and Clinical Oncology Department of Maria Sklodowska-Curie National Research Institute of Oncology, Gliwice Branch, Poland. All were pathologically confirmed with squamous cell carcinoma according to the third edition WHO scale. Forty patients had clinical stage IV, six patients had stage III, according to the seventh edition of the American Joint Committee on Cancer (AJCC), with poor prognostic factors such as heavy smoking and/or heavy-drinking abuse, long-term diagnostic procedures, tumor fragmentation during excision biopsy, difficulties with histopathology diagnosis (2 or more excision biopsies), and rapid progression during diagnostic procedures. The patients aged 22 years or older with primary tumors of the oropharynx, hypopharynx, nasopharynx, and larynx were included for analysis. The patients had biopsy-proven squamous cell carcinoma in the primary and/or cervical lymph nodes. All participants had a performance status of less than 2 according to the ZUBROD WHO scale, good nutritional status, and were inoperable or suitable for organ preservation strategy. Exclusion criteria were the following: distant metastases, squamous cell carcinoma of the nose, sinus, ear, skin, thyroid, salivary, patients with a previous secondary malignancy, performance status greater than or equal to 2 (ZUBROD WHO scale), and no permission for chemotherapy. Due to the high risk of distant failure, multiple involved nodes, a large volume of the primary tumor, and a large volume of nodal disease, patients received iCHT prior to RT or cCHRT (downstaging procedure improving RT planning).

iCHT was realized with one of the following protocols:

(a) Three cycles of TPF administered every 21 days (docetaxel, 75 mg/m^2^ followed by cisplatin 100 mg/m^2^ on day 1, and 5-fluorouracil 1000 mg/m^2^ per day, administered as a continuous 24-h infusion for 4 days) followed by chemoradiotherapy delivered as a sequential therapy—20 patients.
(b) Four cycles of TPF administered every 21 days (docetaxel 75 mg/m^2^ of body-surface area, followed by cisplatin 75 mg/m^2^ on day 1, and 5-fluorouracil 750 mg/m^2^ per day, administered as a continuous 24-h infusion for 4 days) followed by radiotherapy as a sequential therapy—1 patient.
(c) Three cycles of cisplatin plus 5-fluorouracil (PF) administered every 21 days (cisplatin 100 mg/m^2^ on day 1, 5-fluorouracil 1000 mg/m^2^ as a continuous 24-h infusion for 4 days) followed by chemoradiotherapy with cisplatin delivered as a sequential therapy—23 patients.
(d) Three cycles of paclitaxel and carboplatin (PC) administered every 21 days (175 mg/m^2^) and carboplatin at a dose calculated using the Calvert formula area under the curve of 5 followed by chemoradiotherapy with carboplatin delivered as a sequential therapy—2 patients.

The detailed patients’ characteristics are presented in Table 1.

### 4.2. Assessment of Treatment Response to Induction Chemotherapy

The method for assessing the responses to iCHT is described in detail in our previous publication [12]. Briefly, the TNM Classification of Malignant Tumors v.7 was used before and after iCHT as a globally recognized standard for classifying the extent of cancer spread (T: tumor; N: regional involved lymph nodes; and M: distant metastasis). The prefix modifiers “c” and “y” were applied to describe the pre- and post-iCHT stages. In addition, the primary lesions in the head and neck were measured in the images (computed tomography, CT; magnetic resonance, MR; and positron emission tomography–computed tomography, PET–CT) taken before (pre-iCHT) and after induction chemotherapy (post-iCHT) in the radiology department of the Maria Sklodowska-Curie National Research Institute of Oncology. All volumetric measurements were performed by a qualified radiologist using the “MR Segmentation” tool, which is available with Siemens Healthcare syngo.via software, version VB20A (Siemens Healthcare, Berlin, Germany). The lesion volume was calculated and measured in cubic centimeters. The percentage of tumor regression was calculated using the following formula: 100 − (post-iCHT tumor volume) ∗ 100/(preCHT tumor volume).

### 4.3. Blood Serum Samples Collection and Preparation for NMR Spectroscopy

The peripheral venous blood samples were collected before (pre-iCHT) the first cycle of iCHT. The blood samples were incubated for 30 min at room temperature and then centrifuged (1000× *g*, 10 min) to remove a clot and stored frozen at −80 °C. On the day of the NMR measurements, the serum samples were thawed in two steps (at 4 °C and at room temperature) and mixed with phosphate buffer (pH 7.4) containing D2O and trimethylsilylpropanoic acid (TSP), used as an internal reference. The aliquots of 600 µL of the solution were transferred into 5 mm Wilmad WG-1235-7 NMR tubes (Wilmad Labglass, Vineland, NJ, USA) and kept at 4 °C until the NMR analysis.

### 4.4. NMR Measurement Protocol

The same measurement protocol was applied as in our previous metabolomic studies [12]. The 1H NMR spectra were acquired on a Bruker 400 MHz Avance III spectrometer (Bruker BioSpin, Ettlingen, Germany) equipped with a 5 mm PABBI probe. The quality control tests (shim quality, resolution, and water suppression tests) were routinely performed at the beginning of every measurement day, while the acquisition parameters’ optimization (the NMR probe tuning and matching, shimming, determination of the transmitter offset value for the water pulse presaturation, and 90° pulse adjustments) were always adjusted for each sample. The parameters obtained from the quality control tests, as well as the transmitter offset values and the 90° pulse, were compared with the reference parameters for our spectrometer and/or with the archived parameters from previous days and months to make sure that the spectrometer was operating stably.

The receiver gain was set to 90.5 and the temperature to 310 K for all experiments. Four different 1H NMR spectra—NOESY (1D nuclear Overhauser enhancement spectroscopy), CPMG (Carr–Purcell–Meiboom–Gill), diffusion edited (DIFF), and J-resolved (JRES)—were acquired for each serum sample. The pulse sequence parameters are given in the Supplementary Material (Table S1).

### 4.5. NMR Spectra Post-Processing and Metabolite Quantification

The 1D spectra were processed with a line broadening of 0.3 Hz and automatically phase corrected (in TopSpin 3.1pl7 software from Bruker BioSpin, Ettlingen, Germany), referenced to the methyl doublet of alanine at 1.5 ppm, and bucketed over the region 9.0–0.5 ppm with the bucket width set to 0.002 ppm using AMIX 4.0.2 software (Bruker BioSpin, Ettlingen, Germany). The spectrum region of water (5.15–4.38 ppm, d = 0.77 ppm) was removed from the analysis in order to prevent variations in each sample. No normalization was applied.

### 4.6. Metabolite Identification and Quantification

The identification of the metabolites was carried out based on the comparisons with the reference compounds library (in Chenomx NMR Suite 9.0 Professional (Chenomx Inc., Edmonton, AB, Canada)), as well as on the multiplicity and scalar couplings information extracted from the 2D JRES spectra, and using the information from the Human Metabolome Database (http://www.hmdb.ca/ accessed on 6 May 2024) and the available literature.

The low-molecular-weight metabolites were quantified based on the 1D-positive projections of the JRES spectra. The diffusion-edited spectra were used for quantification of the lipid signals. The integrals were measured in the spectral regions defined individually for each metabolite using the “sum all points in region” method in AMIX 4.0.2 (Bruker BioSpin, Ettlingen, Germany) software.

### 4.7. Data Analysis

Due to the small number of samples, the analyses were performed on the quantified metabolites instead of full NMR spectra. The multivariate modeling was conducted using SIMCA v.17 (Sartorius Stedim Data Analytics, Umeå, Sweden) software. Orthogonal partial least squares discriminant analysis (OPLS-DA) was used for class discrimination. In this study, two-class models were used, distinguishing the classes defined as responders and non-responders. A detailed description of these classes is provided in Section 2.1. The quantified NMR metabolites were scaled to unit-variance (UV scaling) because of the large differences in the metabolite integrals. The OPLS-DA models were created using 7-fold cross-validation (4 observations were excluded from the model and were used as a validation group; the process was repeated 7 times with different observations used for each iteration). The univariate statistical analyses were carried out using Statistica 12 software (StatSoft, Hamburg, Germany). The significance threshold was set at 0.05. The Mann–Whitney U (MWU) test (continuous variables) and χ^2^ Chi-squared test (categorical variables) were used to check whether the differences observed between the groups were statistically significant.

The identification of metabolic pathways in which the identified metabolites are involved was performed using MetaboAnalys 6.0 (available at http://www.metaboanalyst.ca accessed on 1 July 2024). The enrichment method was set to Fisher’s exact test, and the topology measure was set to relative-betweenness centrality. The significance threshold for pathway enrichment was set at 0.05 (Holm-adjusted value).

### 4.8. Validation of the Multivariate Models and Receiver Operating Characteristics

The statistical significance of the estimated predictive power of the OPLS-DA models was tested internally in SIMCA v.17 software using permutation testing with the number of permutations doubling the number of the observations in a model (92, 58, and 34 permutations for the study group, males and females, respectively) and ANOVA of the cross-validated residuals (cv-ANOVA). The permutation testing is based on a comparison of the actual model to the number of parallel models based on a fit to randomly reordered class membership in the dataset. The R2Y and Q2 for the permuted models should always be lower than for the actual model [52]. The cv-ANOVA diagnostic formally compares the fit of two models to the same data by the size of their residuals—it tests whether the OPLS-DA model has significantly lower cross-validated predictive residuals than just the variation around the global average [53]. Finally, the significance of the identified metabolites is assessed with the univariate MWU test.

The true positive classification rates (TPR) for the ROC curves were calculated based on the OPLS-DA misclassification tables. Each ROC plot (Figure 1) presents TPR for the responders (>75) and non-responders (<75), respectively.

## 5. Conclusions

This retrospective metabolomics study shows that a serum 1H NMR spectroscopy can potentially be a useful tool for predicting the response to iCHT in HNSCC.

A number of metabolites distinguishing the male responders from the non-responders have been identified. These are serum lipids, amino acids involved in protein synthesis and degradation (glycine, glutamate, alanine, and isoleucine), ketone bodies (indicators of nutritional status), formate (metabolite correlated with vitamin B12 deficiency), and N-acetylcysteine (which, together with glycine, is involved in protection against oxidative damage and increasing the ability to scavenge oxidants).

While the relationship between the lipid compounds and chemotherapy is still unclear, other results indicate that the nutritional status, the capacity of the immune system, and the efficiency of metabolism related to protein synthesis may be prognostic factors for the response to induction chemotherapy in males with HNSCC.

These preliminary results need to be evaluated in a larger group with an external test set.

## Figures and Tables

**Table 1:** Characteristics of the study group.

	Study Group				Males				Females			
	Responders		Non-Responders		Responders		Non-Responders		Responders		Non-Responders	
Patients	27		19		19		10		8		9	
Age (median)	57		54.5		58		58.5		52.5		53	
Age (range)	22–71		40–73		22–65		51–73		34–71		40–60	
Primary tumor site												
Oropharynx	13		5		9		4		5		0	
Nasopharynx	4		7		2		3		2		4	
Hypopharynx	3		6		3		2		0		4	
Larynx	6		2		5		1		1		1	
	T stage				T stage				T stage			
	**c**	**y**	**c**	**y**	**c**	**y**	**c**	**y**	**c**	**y**	**c**	**y**
0	0	11	0	0	0	10	0	0	0	1	0	0
1	2	11	3	11	1	7	2	6	0	5	1	4
2	9	4	3	5	7	2	2	3	2	2	1	2
3	9	0	7	1	7	0	3	0	2	0	4	1
4	6	0	7	3	3	0	3	1	4	0	3	2
	N stage				N stage				N stage			
	**c**	**y**	**c**	**y**	**c**	**y**	**c**	**y**	**c**	**y**	**c**	**y**
0	1	9	2	8	0	7	0	4	1	3	2	3
1	3	4	4	4	2	2	2	3	1	2	2	1
2a	0	2	5	4	2	1	3	1	3	1	2	3
2b	10	5	5	3	4	5	3	2	1	0	1	1
2c	9	5	3	2	5	3	1	1	1	2	2	1
3	8	0	1	0	6	0	1	0	1	0	0	0
	TNM stage				TNM stage				TNM stage			
	**c**	**y**	**c**	**y**	**c**	**y**	**c**	**y**	**c**	**y**	**c**	**y**
0	0	3	0	0	0	3	0	0	0	0	0	0
I	0	7	0	6	0	5	0	4	0	3	0	1
II	0	0	0	3	0	0	0	1	0	0	0	2
III	2	5	4	4	1	2	1	2	1	3	3	2
IVa	18	10	15	8	13	7	19	4	6	2	6	4
IVb	6	0	1	0	5	1	0	0	1	0	0	0

**Table 2:** The results from the OPLS-DA and statistical analyses.

OPLS-DA Model Diagnostics										
		Study Group			Males			Females		
Predictive component		R2X	R2Y	Q2	R2X	R2Y	Q2	R2X	R2Y	Q2
		0.29	0.30	0.20	0.19	0.59	0.37	0.33	0.33	0.12
Orthogonal component		R2X(o)			R2X(o)			R2X(o)		
		-			0.23			-		
cv-ANOVA *p*		0.008			0.02			0.42		
List of the important metabolites from the OPLS-DA model										
	Name	p(corr)	*p*-value	Median FC	p(corr)	*p*-value	Median FC	p(corr)	*p*-value	Median FC
Metabolites increased in responders										
1	Isoleucine 0.95 ppm	**0.77**	**0.03**	1.09	**0.56**	**0.04**	1.30	**0.48**	0.90	0.91
2	Isoleucine 1.02 ppm	**0.76**	**0.01**	1.20	**0.52**	**0.04**	1.25	**0.50**	0.47	1.10
3	Alanine	**0.57**	**0.01**	1.23	**0.55**	**0.05**	1.28	**0.38**	0.09	1.26
4	Glycine	**0.11**	**0.07**	1.14	**0.38**	**0.04**	1.27	**0.40**	0.13	1.11
5	Tyrosine	**0.62**	**0.02**	1.19	**0.53**	**0.07**	1.23	**0.52**	0.1	1.14
6	N-acetylcysteine	**0.30**	**0.01**	1.13	**0.54**	**0.01**	1.13	**0.39**	0.42	1.11
7	Lipids 0.9 ppm	**0.44**	0.15	1.05	**0.36**	**0.02**	1.12			
8	Lipids 1.3 ppm	**0.52**	0.17	1.06	**0.36**	**0.07**	1.13			
9	Lipids 5.3 ppm	**0.46**	0.17	1.02	**0.36**	**0.02**	1.09			
10	Acetate							**0.53**	0.16	1.26
14	Formate							**0.66**	0.96	1.08
Metabolites decreased in responders										
10	Acetate	−0.26	0.79	0.95	**−0.57**	0.28	0.85			
11	Glutamate	**−0.66**	0.24	0.91	**−0.30**	0.81	0.98	**−0.76**	0.42	0.68
12	3-hydroxybutyrate 4.21	**−0.72**	–	0.92	**−0.33**	0.15	0.97	**−0.80**	0.06	0.66
13	3-hydroxybutyrate 1.23	**−0.66**	0.10	0.67	−0.23	0.58	0.91	**−0.76**	0.27	0.55
14	Formate	−0.23	0.18	0.96	**−0.56**	0.20	0.95			
15	Acetone	**−0.38**	0.95	1.01	−0.28	0.77	1.02	**−0.51**	0.96	1.02
16	Acetoacetate	**−0.52**	0.33	0.85	−0.25	0.84	1.01	**−0.63**	0.53	1.02
7	Lipids 0.9 ppm							**−0.61**	0.60	0.99
8	Lipids 1.3 ppm							**−0.41**	0.89	1.03
9	Lipids 5.3 ppm							**−0.56**	0.81	0.96

Legend: The results take into account the resonance signals of the functional groups of a given metabolite at different chemical shifts, e.g., isoleucine at 0.95 and 1.02 ppm, lipids at 0.9, 1.3, and 5.3 ppm, as well as 3-hydroxybutyrate at 1.23 and 4.21 ppm. The p(corr) values > 0.3 and *p*-values < 0.05 thresholds are bolded.

## Data Availability

The datasets generated and/or analyzed during the current study are available from the corresponding author upon reasonable request.

## Supplementary Materials

The following supporting information can be downloaded at: https://www.mdpi.com/article/10.3390/ijms25147555/s1, Table S1. The characteristics of the acquired spectra as well as the pulse sequence parameters.
