# Supplementary material for: NMR-Based Metabolomics of Blood Serum in Predicting Response to Induction Chemotherapy in Head and Neck Cancer—A Preliminary Approach
Source: Int J Mol Sci. 2024 Jul 10;25(14):7555. doi: 10.3390/ijms25147555 (PMC11277221; doi:10.3390/ijms25147555)
Supplement: Supplementary file 1 [file ijms-25-07555-s001.zip › ijms-3072263-supplementary.pdf]

Supplementary material for:

NMR-Based Metabolomics of Blood Serum in Predicting Response to Induction Chemotherapy in Head and Neck Cancer–A Preliminary Approach

Łukasz Boguszewicz [1,*], Agata Bieleń [2], Jarosław Dawid Jarczewski [3], Mateusz Ciszek [1], Agnieszka Skorupa [1], Jolanta Mrochem-Kwarciak [4], Krzysztof Składowski [2] and Maria Sokół [1]

1 Department of Medical Physics, Maria Sklodowska-Curie National Research Institute of Oncology, Gliwice Branch, 44-102 Gliwice, Poland; lukasz.boguszewicz@gliwice.nio.gov.pl (Ł.B), mateusz.ciszek@gliwice.nio.gov.pl (M.C.), agnieszka.skorupa@gliwice.nio.gov.pl (A.S.), maria.sokol@gliwice.nio.gov.pl (M.S.)
2 1st Radiation and Clinical Oncology Department, Maria Sklodowska-Curie National Research Institute of Oncology, Gliwice Branch, 44-102 Gliwice, Poland; agata.bielen@gliwice.nio.gov.pl (A.B.), krzysztof.skladowski@gliwice.nio.gov.pl (K.S.)
3 Radiology and Diagnostic Imaging Department, Maria Sklodowska-Curie National Research Institute of Oncology, Gliwice Branch, 44-102 Gliwice, Poland; jaroslaw.dawid.jarczewski@gmail.com (J.D.J)
4 Analytics and Clinical Biochemistry Department, Maria Sklodowska-Curie National Research Institute of Oncology Gliwice Branch, 44-102 Gliwice, Poland; jolanta.mrochem-kwarciak@gliwice.nio.gov.pl (J.M-K.)
* Correspondence: lukasz.boguszewicz@gliwice.nio.gov.pl (Ł.B)

The characteristics of the acquired spectra as well as the pulse sequence parameters

- NOESY (Nuclear Overhauser Effect Spectroscopy) – an overview of all types of molecules.
- CPMG (Carr-Purcell-Meiboom-Gill) – an information on only low molecular weight metabolites.
- DIFF (diffusion edited) - mainly macromolecular signals.
- Two dimensional (2D) JRES (J-resolved) – a visualization of scalar couplings and improved metabolite identification.

Table S1. NMR pulse sequence parameters.

Pulse program	**NOESYGPPR1D**	**CPMGPR1D**	**LEDBPGPPR2S1D**	**JRESGPPRQF**
**TD**	65536	65536	65536	8192
**SW** [ppm]	30	20	30	16.62
**AQ** [sec]	2.73	4.09	2.73	0.62
**D1** [sec]	4	4	4	2
**D8** [sec]	0.01	-	-	-
**D16** [sec]	-	-	0.0002	0.0002
**D20** [sec]	-	0.0003	0.12	-
**D21** [sec]	-	-	0.005	-
**DS**	4	4	4	16
**L4**	-	126	-	-
**NS**	32	64	64	1
**DELTA1** [sec]	-	-	0.11572488	-
**DELTA2** [sec]	-	-	0.004172	-
